# Esketamine Treatment and Its Impact on Suicidality in Treatment-Resistant Depression: A Narrative Review

**DOI:** 10.1192/j.eurpsy.2026.12071

**Published:** 2026-07-31

**Authors:** I. C. Mândraș, A. L. Comșa

**Affiliations:** 1 Psychology and Educational Sciences, Babes-Bolyai University; 2 Psychiatry, County Clinical Emergency Hospital, Cluj-Napoca , Romania

## Abstract

**Introduction:**

Suicidality represents a major clinical and public health challenge, particularly among patients with treatment-resistant depression (TRD), who carry a disproportionately high risk of suicide. Traditional antidepressants often have delayed onset and limited efficacy in reducing suicidal ideation in this high-risk population, highlighting the need for rapid-acting interventions. Esketamine, a rapid-acting NMDA receptor antagonist, has shown potential for reducing suicidal ideation, but existing evidence remains heterogeneous, and uncertainties persist regarding its mechanisms, long-term safety, and applicability across diverse patient populations.

**Objectives:**

The objective of this review was to evaluate the impact of esketamine on suicidality in patients with treatment-resistant depression, to identify existing research gaps, and to outline potential future directions.

**Methods:**

We searched PubMed for articles published between January 2020 and September 2025 using the string ("esketamine"[MeSH Terms] OR “esketamine”[All Fields]) AND ("suicid*"[All Fields] OR “suicidal ideation”[MeSH Terms] OR “suicidal”[All Fields]) AND ("treatment-resistant depression"[MeSH Terms] OR “treatment-resistant depression”[All Fields] OR “TRD”[All Fields]). The search yielded 91 records; studies reporting suicidality outcomes in treatment-resistant depression were included, while case reports and studies without suicidality outcomes were excluded.

**Results:**

Esketamine demonstrates rapid reduction of suicidal ideation in patients with treatment-resistant depression, often observable within hours of intranasal administration. Acute adverse events are generally manageable within supervised certified settings. Structured induction and maintenance protocols support sustained symptom improvement, though patient responses remain heterogeneous. Integration into clinical practice benefits from multidisciplinary support, psychosocial interventions, and ongoing monitoring of somatic health. Operational and logistical challenges, including clinic workflow, cost, and access in resource-limited settings, continue to affect real-world implementation. Remaining research gaps involve standardized suicidality assessment, mechanistic elucidation, long-term safety, predictive biomarkers, and the inclusion of underrepresented populations.

**Conclusions:**

Esketamine offers a rapid-acting pharmacological option to mitigate suicidal ideation in treatment-resistant depression, bridging critical high-risk intervals where conventional antidepressants may be insufficient. While short-term safety and efficacy are supported, significant research gaps remain. Addressing these gaps will be essential to refine patient selection, optimize maintenance strategies, and ensure equitable access. Esketamine’s integration into suicide prevention frameworks holds promise as a targeted intervention to interrupt suicidal trajectories.

**Disclosure of Interest:**

None Declared

